# Transcriptome and molecular regulatory mechanisms analysis of gills in the black tiger shrimp *Penaeus monodon* under chronic low-salinity stress

**DOI:** 10.3389/fphys.2023.1118341

**Published:** 2023-03-01

**Authors:** Yun-Dong Li, Meng-Ru Si, Shi-Gui Jiang, Qi-Bin Yang, Song Jiang, Li-Shi Yang, Jian-Hua Huang, Xu Chen, Fa-Lin Zhou, ErChao Li

**Affiliations:** ^1^ Key Laboratory of Tropical Hydrobiology and Biotechnology of Hainan Province, Hainan Aquaculture Breeding Engineering Research Center, College of Marine Sciences, Hainan University, Haikou, China; ^2^ Key Laboratory of South China Sea Fishery Resources Exploitation and Utilization, Ministry of Agriculture and Rural Affairs, South China Sea Fisheries Research Institute, Chinese Academy of Fishery Sciences, Guangzhou, China; ^3^ Key Laboratory of Efficient Utilization and Processing of Marine Fishery Resources of Hainan Province, Sanya Tropical Fisheries Research Institute, Sanya, China; ^4^ Hainan Yazhou Bay Seed Laboratory, Sanya, China

**Keywords:** *Penaeus Monodon*, Chronic low-salinity stress, transcriptome, osmoregulation, adaptation mechanisms

## Abstract

**Background:** Salinity is one of the main influencing factors in the culture environment and is extremely important for the survival, growth, development and reproduction of aquatic animals.

**Methods:** In this study, a comparative transcriptome analysis (maintained for 45 days in three different salinities, 30 psu (HC group), 18 psu (MC group) and 3 psu (LC group)) was performed by high-throughput sequencing of economically cultured *Penaeus monodon*. *P. monodon* gill tissues from each treatment were collected for RNA-seq analysis to identify potential genes and pathways in response to low salinity stress.

**Results:** A total of 64,475 unigenes were annotated in this study. There were 1,140 upregulated genes and 1,531 downregulated genes observed in the LC vs. HC group and 1,000 upregulated genes and 1,062 downregulated genes observed in the MC vs. HC group. In the LC vs. HC group, 583 DEGs significantly mapped to 37 signaling pathways, such as the NOD-like receptor signaling pathway, Toll-like receptor signaling pathway, and PI3K-Akt signaling pathway; in the MC vs. HC group, 444 DEGs significantly mapped to 28 signaling pathways, such as the MAPK signaling pathway, Hippo signaling pathway and calcium signaling pathway. These pathways were significantly associated mainly with signal transduction, immunity and metabolism.

**Conclusions:** These results suggest that low salinity stress may affect regulatory mechanisms such as metabolism, immunity, and signal transduction in addition to osmolarity in *P. monodon*. The greater the difference in salinity, the more significant the difference in genes. This study provides some guidance for understanding the low-salt domestication culture of *P. monodon*.

## 1 Introduction

The stress response is a complex physiological mechanism activated by stimuli that an organism perceives as potentially threatening (Vallejos-Vidal et al., 2022). It involves the sum of continuous and coordinated activation mechanisms at different functional levels, including molecular processes that have a direct impact on gene expression and protein synthesis, and will ultimately activate responses from the cellular to the systemic and performance levels (Parra et al., 2015). Salinity is one of the key factors regulating the distribution, abundance and diversity of aquatic animals and is one of the main environmental factors exerting selection pressure on aquatic animals (Sun et al., 2020). Changes in environmental salinity can directly affect the composition of aquatic animal fluids and the homeostasis of the internal environment (Chen et al., 2015). It has been shown that salinity stress can alter the diffusion of salts between the hemolymph and the environment, leading to altered cellular states. Within the critical point salinity range, significant changes in protein function and structure as well as in the physicochemical properties of NaCl solutions can occur (Normant et al., 2005). Low salinity alters the cell membrane potential, the optical density of solubilized proteins and the activity of various enzymes (Esparza-Leal et al., 2019). Prolonged hyposalinity exposure affects the physiology of the organism, reducing the immune response of aquatic animals to attack by external factors and leading to reduced survival (Chen and He, 2019). Under moderate hypoosmotic stress, aquatic animals typically meet the increased cost of osmoregulation by increasing their regular metabolic rate or reallocating energy to osmoregulatory processes (Rivera-Ingraham et al., 2016). Under extreme hypoosmotic stress, many aquatic animals are limited to maintaining basic functions to conserve energy reserves until the environment improves (Podbielski et al., 2022). Therefore, elucidating the adaptation mechanisms of aquatic animals to different salinities is crucial for the continued health of aquaculture.

*Penaeus monodon* (also known as black tiger shrimp) is one of the most commercially valuable species of aquaculture shrimp (Li et al., 2018; Li et al., 2020). *P. monodon* is popular with farmers and consumers due to its high economic and nutritional value (FAO, 2018). *P. monodon* has a wide range of salinity adaptations and can grow in a wide range of salinities from one psu ∼57 psu (Joseph and Philip, 2020). *P. monodon* has an isosmotic point of 750 mOsm·kg^-1^ (equivalent to 25 psu). In recent years, its culture has advanced significantly from coastal marine waters to inland low-salinity waters due to mariculture disease outbreaks and near-coastal pollution (Vezzulli, 2022). Therefore, understanding the mechanisms of *P. monodon* adaptation in long-term low-salt stress will be beneficial to its healthy culture. The regulatory mechanisms of various genes in salinity adaptation of *P. monodon* have been reported, including cold shock domain containing protein E1 (Si et al., 2022a), ecdysone-induced protein E74 (Si et al., 2022b), calreticulin, calnexin and endoplasmic reticulum protein 57 (Visudtiphole et al., 2017). However, systematic studies on the adaptation mechanisms of *P. monodon* in long-term low-salt stress environments are still lacking.

With the development of high-throughput sequencing technologies, RNA-seq offers the opportunity to study the mechanisms of transcriptional variation in organisms under different environmental conditions (de Jong et al., 2019). Transcriptome sequencing not only allows the detection of almost all validated genes expressed in a specific cell or organ but also allows a comprehensive resolution of the regulatory mechanisms involved in a specific biological process based on the structure and function of the differential genes (Stupnikov et al., 2021). In addition, transcriptome sequencing allows the simultaneous analysis of all physiological processes, including metabolism (Xiao et al., 2020), proteostasis (Li et al., 2022), osmoregulation (Gao et al., 2021) and other cellular processes (Ge et al., 2022). Thus, transcriptome technologies address key gene expression patterns in non-model organisms in specific environments and enable the detection of unknown genes and the discovery of novel transcripts. In recent years, transcriptome technologies have been widely used for transcriptome assembly and annotation in many aquatic animals, such as *Macrobrachium rosenbergii* (Liu X. et al., 2021), *Coilia nasus* (Gao et al., 2021) and *Penaeus vannamei* (Yu et al., 2022). Thus, RNA-seq is expected to make an important contribution to the resolution of long-term low salinity adaptation mechanisms in *P. monodon*.

The gill is an important respiratory organ of *P. monodon* and is involved in its respiratory metabolism, acid-base balance, ammonia waste excretion, ion transport and osmotic pressure regulation (Henry et al., 2012). Therefore, in this study, the gene expression profiles of *P. monodon* gill tissues reared at three salinity levels were examined by RNA-seq. The results of this study will provide new insights into the mechanisms of osmotic pressure regulation in *P. monodon* under chronic low salinity stress.

## 2 Materials and methods

### 2.1 Ethical statement

Experimental shrimp were approved by the Animal Care and Use Committee of the Chinese Academy of Fisheries Sciences (CAFS) of the South China Sea Fisheries Research Institute of the Chinese Academy of Fisheries Science (NO: SCSFRI96-253). Our experiments were implemented in accordance with national and institutional guidelines for the protection and use of CAFS laboratory animals.

### 2.2 Experimental animals

Healthy *P. monodon* were obtained from the Shenzhen base of the South China Sea Fisheries Research Institute of the Chinese Academy of Fisheries Science. The experimental *P. monodon* were 7.8 ± 0.8 cm in length and 7.3 ± 1.0 g in body mass. The experiments were carried out in concrete ponds at a salinity of approximately 30 psu, 27–28°C and pH 7.6–7.8 for 1 week prior to the start of the experiment. During this period, the ponds were aerated and fed commercial bait four times a day. One-third of the water volume was replaced daily using multistage filtered and disinfected seawater.

### 2.3 Experimental methods

#### 2.3.1 Low salinity stress trials and sampling procedures

The experiment was divided into three groups: a pure seawater group with a salinity of 30 psu (high-salinity challenge, HC), a group with a salinity of 18 psu (moderate-salinity challenge, MC), and a group with a salinity of 3 psu (low-salinity challenge, LC). The different salinities required for the experiments were adjusted by mixing bay water disinfected by multistage sand filtration with tap water in proportions, and the salinity was measured using a high-precision salinometer (ATAGO, Guangzhou). The formal experiments were carried out in special buckets for shrimp culture with a volume of 500 L and 400 L of culture water. Four parallels were set up in each group, using one bucket per parallel. Each bucket contained 60 juvenile shrimp. The salinities used were all 18 psu at the beginning and then adjusted by 3 psu each day until each group reached the desired salinity of 30 psu, 18 psu and 3 psu, respectively, before the official start of the culture trials at the three salinities. For 45 days of culture, water quality was tested daily, and shrimp growth and feeding were observed. Feeding was performed 3 times a day, weighing the same bait and feeding regularly. Water was changed completely every 3 days to maintain the water quality. At the end of the culture, gill tissue samples were taken from each of the three groups for transcriptome sequencing. Every three shrimp from the same group were mixed into one tube. All tissues were immediately frozen in liquid nitrogen and stored at −80°C until RNA extraction.

#### 2.3.2 RNA extraction, library construction and sequencing

Total RNA was extracted from each sample of *P. monodon* gill tissue according to the instructions of the mirVana™ miRNA ISOlation Kit (Ambion, America). The product (concentration ≥50 ng mL^−1^) was examined by 1.5% agarose gel electrophoresis for completeness, which showed clear 28S and 18S bands with no obvious dragging. RNA purity was measured by a NanoDrop 2000 (Thermo, America) with A260/280 ≈ 2.0. A total of 4 μg of total RNA was used to construct RNA-seq libraries according to the instructions of the TruSeq Stranded mRNA LTSample Prep Kit (Illumina, America) (Loureiro et al., 2019). The RNA libraries were tested by an Agilent 2100 microarray for length and quality. After passing quality control, all RNA libraries were sequenced using an Illumina HiSeqTM 2500 instrument (Illumina, United States). The raw reads were archived in the sequence read archive of the National Center for Biotechnology Information under accession number SRP262105.

#### 2.3.3 De novo assembly and annotation

Transcript sequences were obtained using the paired-end splicing method with Trinity trinityrnaseq_r20131110 software (Grabherr et al., 2011), and the final unigene was obtained using TGICL 2.1 software (Pertea et al., 2003) for clustering and deredundancy extension to obtain the final unigene.

Unigene sequences were aligned by blastx to non-redundant (NR), Swiss-Prot protein (Swiss-Prot), Clusters of orthologous groups for eukaryotic complete genomes (KOG), Kyoto encyclopedia of genes and genomes (KEGG) and gene ontology (GO) libraries for comparison. The annotations with e < 1e^−5^ were taken to obtain the protein with the highest sequence similarity to the given Unigene. The KEGG annotation information of UniGene was obtained by using KAAS (http://www.genome.jp/kaas-bin/kaas_main), and based on the annotation results of SWISSPROT, the GO term was mapped to obtain the GO protein function annotation information of the UniGene. The annotation summary table information was obtained by merging Unigene with the best one from each database.

#### 2.3.4 Differentially expressed gene analysis and functional enrichment analysis

The expression levels of all DEGs were quantified based on the negative binomial distribution test in DESeq software (http://bioconductor.org/packages/release/bioc/html/DESeq.html). The NB (negative binomial distribution test) was used to test the significance of differences in the number of reads, and baseMean values were used to estimate the amount of gene expression.

The number of differential genes included in each GO entry was counted, and the significance of differential gene enrichment in each GO entry was calculated using the hypergeometric distribution test. A *p*-value <0.05 and log_2_-fold change value >2 indicated that differential genes were enriched in that GO entry.

## 3 Results

### 3.1 Transcriptome sequencing and *de novo* assembly

To investigate the dynamic mRNA expression levels of *P. monodon* under different salinity stresses, mRNA-seq libraries were constructed from nine samples and sequenced by the Illumina NovaSeq platform. These libraries were HC1, HC2, HC3, MC1, MC2, MC3, LC1, LC2 and LC3. Nine libraries were obtained with approximately 49,236,624, 48,478,994, 48,743,030, 47,468,446, 48,525,278, 48,485,970, 48,220,504, 47,663,910 and 47,972,088 clean reads with average GC contents of 45.5%, 45%, 44%, 45.5%, 45.5%, 45%, 45%, 46%, and 45%, respectively. The samples were tested with Q30 values higher than 94.00%, indicating a good quality of transcriptome sequencing (Table 1). Due to the lack of a reference genome for *P. monodon* transcriptome analysis, the Trinity method was used to assemble high-quality reads. A total of 64,475 unigenes were obtained from transcriptome sequencing. The maximum length was 38,289 bp, the average length was 1,275.72 bp, and the N50 was 2,158 bp. Among these unigenes, 37,266 (36.88%) were longer than 1,000 bp, and the 300–500 bp and 500–1,000 bp unigenes were 21,796 (33.81%) and 18,898 (29.31%), respectively.

**TABLE 1:** Overview of the sequencing results.

Sample	Raw reads	Clean reads	Valid bases (%)	Q30 (%)	GC (%)
HC1	50,738,124	49,236,624	97.00	94.45	45.50
HC2	50,070,750	48,478,994	96.78	94.50	45
HC3	50,471,830	48,743,030	96.53	94.10	44
MC1	49,304,948	47,468,446	96.23	94.01	45.50
MC2	50,386,986	48,525,278	96.26	94.11	45.50
MC3	50,217,760	48,485,970	96.51	94.34	45
LC1	49,842,448	48,220,504	96.71	94.36	45
LC2	49,281,406	47,663,910	96.68	94.24	46
LC3	49,409,288	47,972,088	97.05	94.61	45

### 3.2 Functional annotation and classification of *P. monodon* transcripts

A total of 64,475 unigenes were annotated in five commonly used databases, including NR, Swiss-Prot, KOG, KEGG and GO. E-values less than 1e^−60^ were present in 55.02% of the 20,990 unigenes (32.56%) annotated to the NR database. When mapped to other species, 10,780 unigenes (27.75%) showed a preference for sequence similarity to Hyalella azteca species. Sequences from 472 unigenes (2.25%) showed similarities to *P. monodon* species. Due to the shortage of decapoda genomic resources, up to 51.36% and 26.65% of unigenes could not be annotated in the NR and Swiss-Prot databases, respectively, and may represent the genetic specificity of *P. monodon*.

### 3.3 Simple sequence repeat (SSR) analysis

The 64,475 unigenes (total sequence length 82,251,807bp) were examined using MISA and Primmer 3 software, and their SSR loci were analyzed. We identified 51,544 SSR loci (31.70% of the total unigenes), of which 12,634 sequences contained more than one SSR. Among all SSRs, 5,387 SSRs were present in compound formation. Among them, the most represented repeat type was mononucleotide (46.04%), and the least represented repeat types were pentanucleotide and hexanucleotide (0.17%) (Table 2).

**TABLE 2:** Repeat type frequency of SSRs in the tested *P. monodon* transcriptome.

Repeats	5	6	7	8	9	10	11	>11	Total	%
1 bp	0	0	0	0	0	9,207	4,273	10,250	23,730	46.04
2 bp	0	5,161	3,289	2,645	3,163	2,909	898	85	18,150	35.21
3 bp	4,044	2,361	1,576	147	4	2	1	6	8,141	15.79
4 bp	1,278	129	9	2	1	1	2	11	1,433	2.78
5 bp	23	3	3	1	2	0	0	6	38	0.07
6 bp	14	5	5	5	1	4	8	10	52	0.10
total	5,359	7,659	4,882	2,800	3,171	12,123	5,182	10,368	51,544	100
%	10.40%	14.86%	9.47%	5.43%	6.15%	23.52%	10.05%	20.11%	100%	—

### 3.4 Differentially expressed gene screening

**FIGURE 1:**
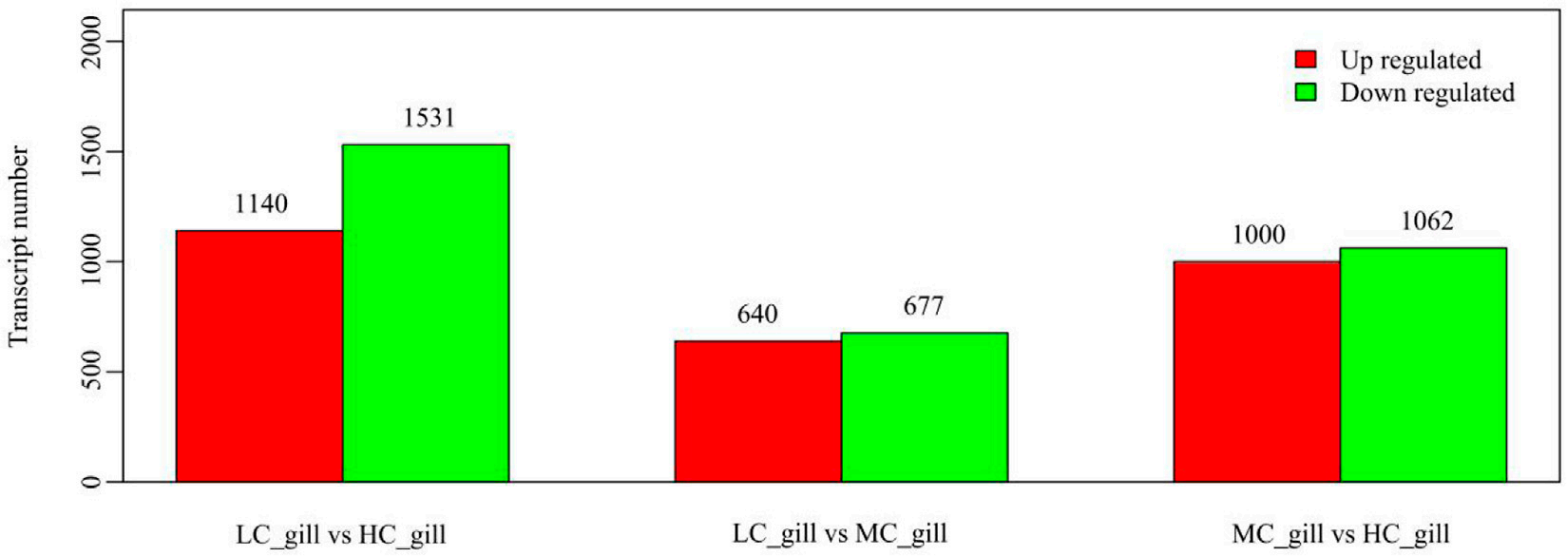
The number of differentially expressed genes in the three groups.

The genes responsive to low salinity stress and the preliminary mechanism of *P. monodon* adaptation to low salinity stress were screened. The expression levels were calculated and compared between the three groups (HC, MC and LC groups), and the DEGs of the MC and LC groups compared to the HC group were determined (the conditions for significant differences were *p*-value <0.05 and difference multiplicity >2). The DEG results showed that compared to the HC group, 1,000 upregulated genes and 1,062 downregulated genes were observed in the MC group, and 1,140 upregulated genes and 1,531 downregulated genes were observed in the LC group compared to the HC group (Figure 1).

**FIGURE 2:**
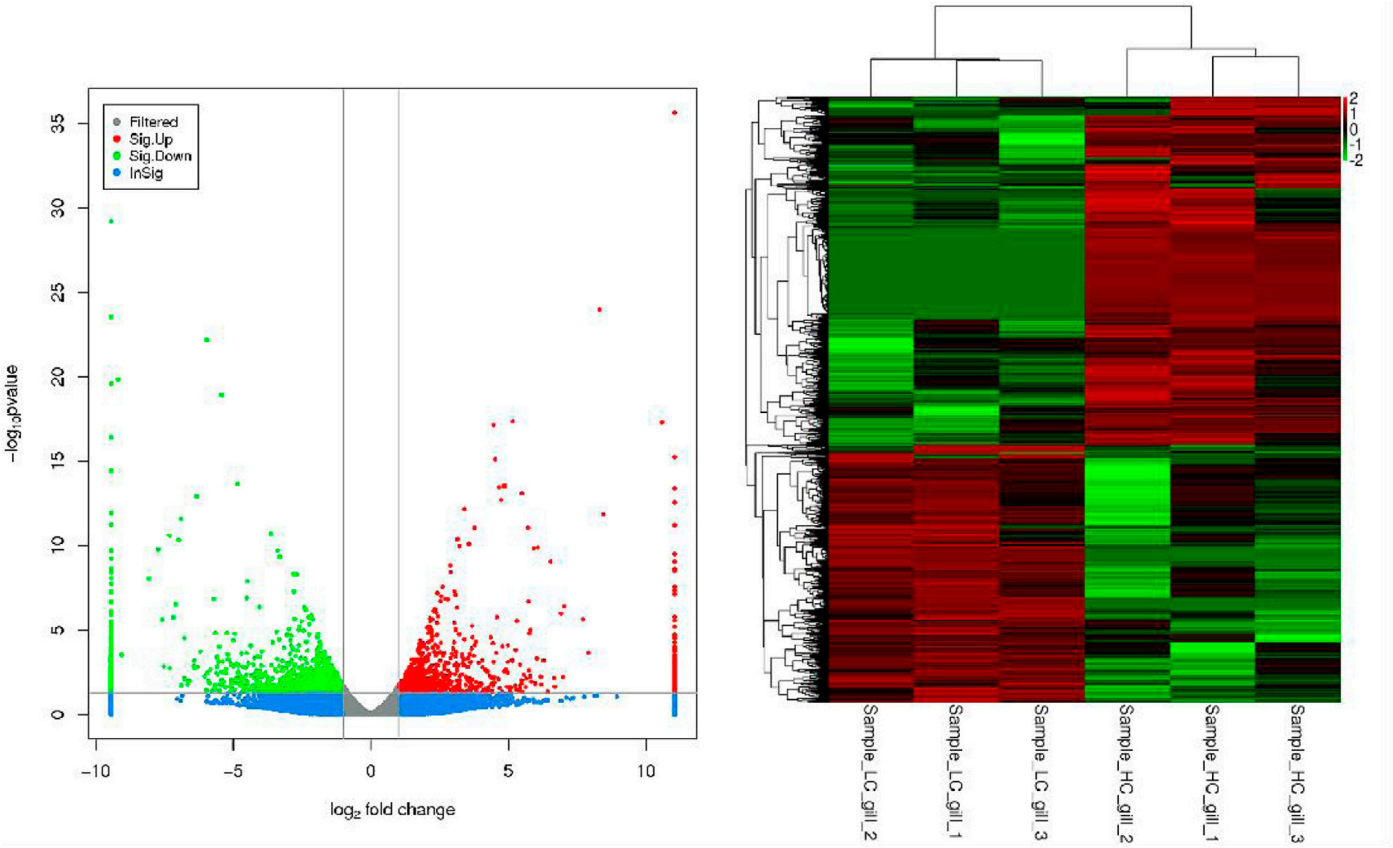
Cluster analysis results for each difference grouping (LC vs. HC).

Figure 2 shows the grouped clustering analysis of differentially expressed genes in three salinity groups, HC, MC and LC. The results showed that the differentially expressed genes basically had a clear up- and downregulation relationship in the two samples. The genes thus classified are good references for conducting gene enrichment analysis and the resolution of salinity adaptation mechanisms.

### 3.5 GO and KEGG enrichment analysis of DEGs

**FIGURE 3:**
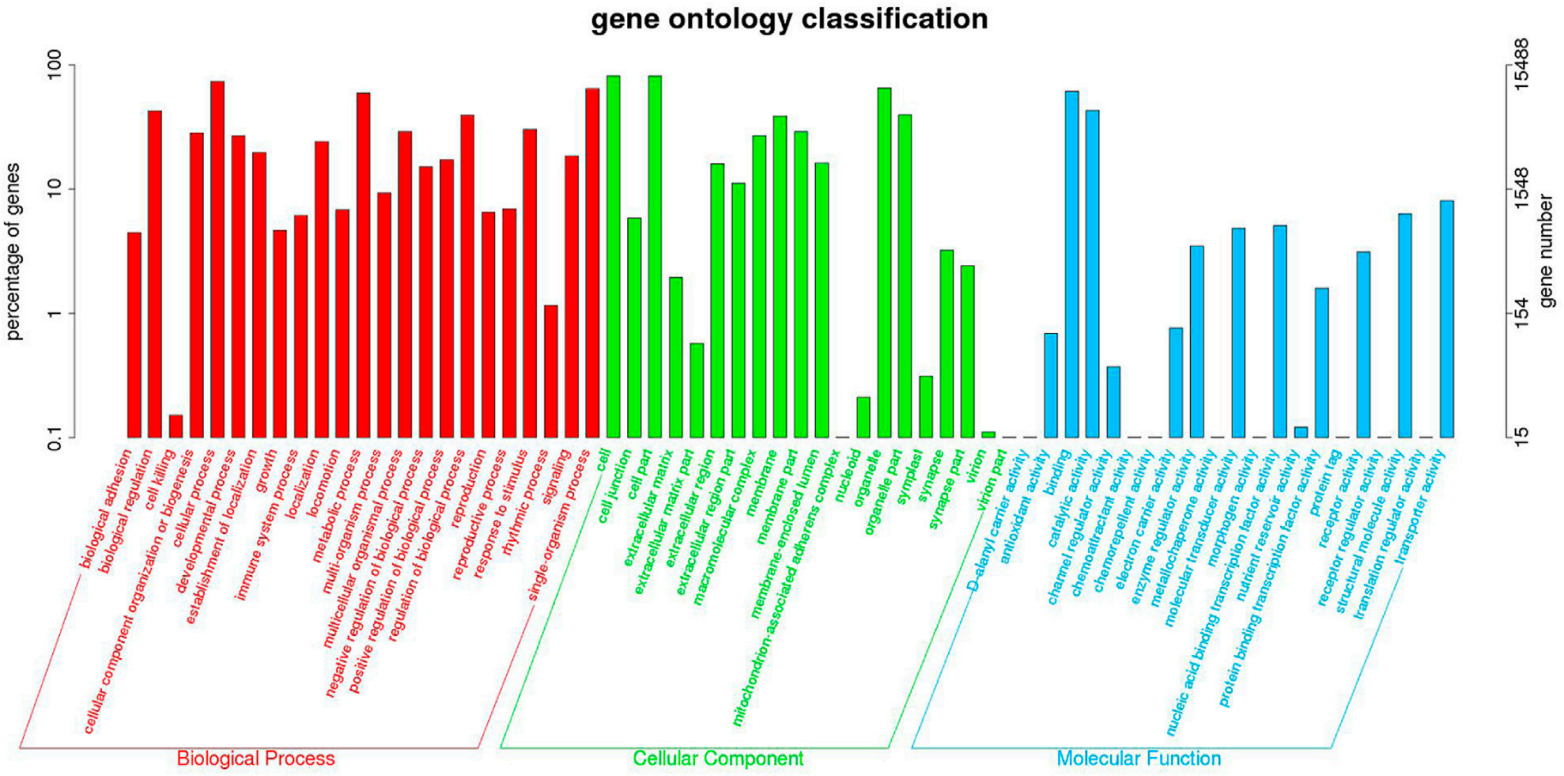
GO Level 2 classification statistics results of DEGs.

**FIGURE 4:**
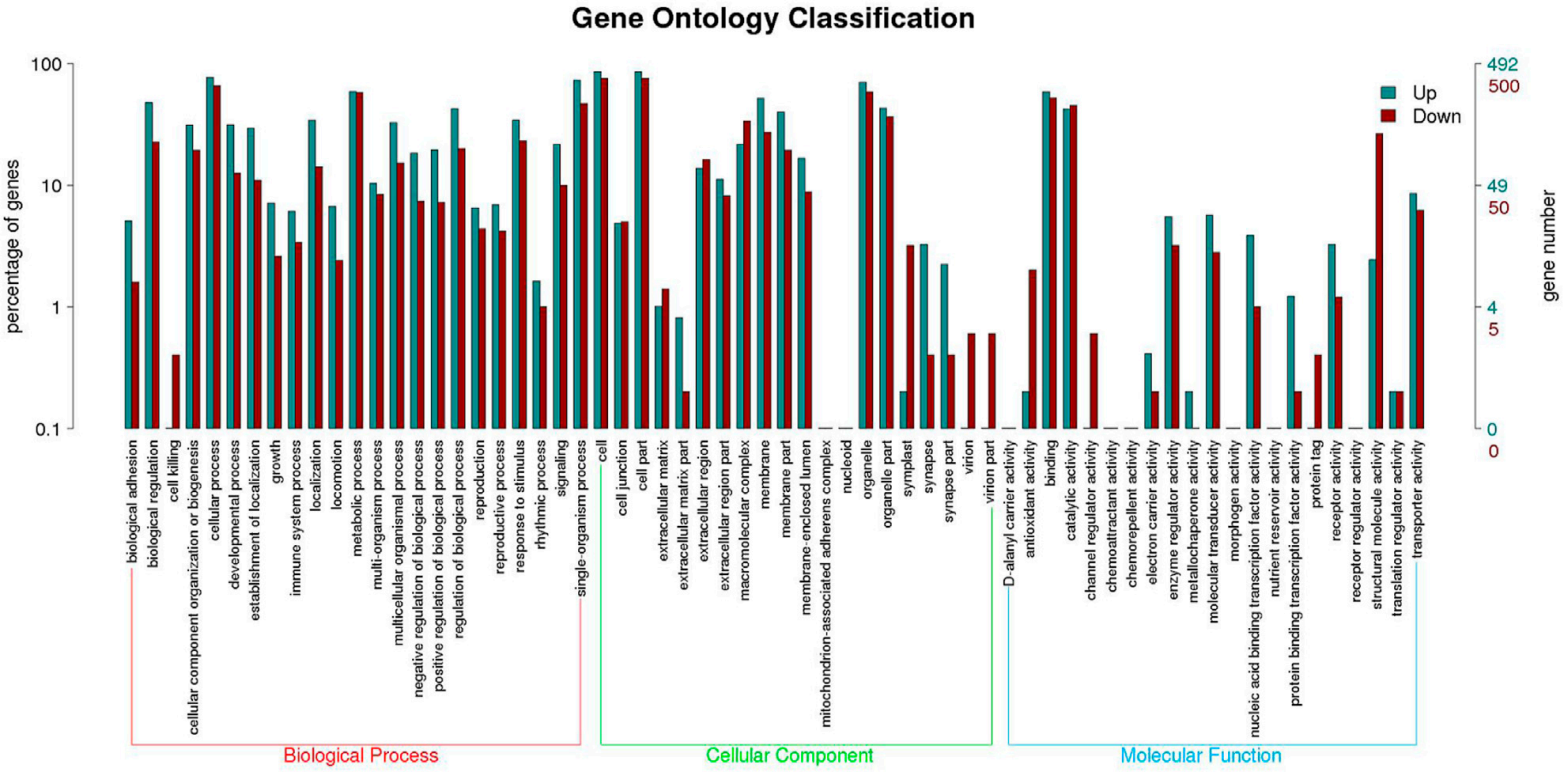
GO Level 2 classification statistics results of up- and downregulated differentially expressed genes.

Functional annotation analysis of DEGs was used to further compare and explore the transcriptome regulatory patterns under different salt concentration stresses. Figure 3 shows the GO enrichment analysis of all differentially expressed genes. The GO enrichment results showed the highest enrichment (*p*-value <0.05) in the three clusters of biological processes, molecular functions and cellular components. Figure 4 shows the integration of GO Level 2 classification of upregulated genes and downregulated genes in the differential grouping. The results showed that the differentially expressed genes were more annotated in the biological process category in cellular process, single-organism process, metabolic process, biological regulation, regulation of biological process and other functional localizations. The enriched cellular components were mainly functions such as cell, cell part, organelle, organelle part and membrane. The enrichment in molecular function was mainly in the functional classification of binding, catalytic activity, structural molecule activity, and transporter activity.

**FIGURE 5:**
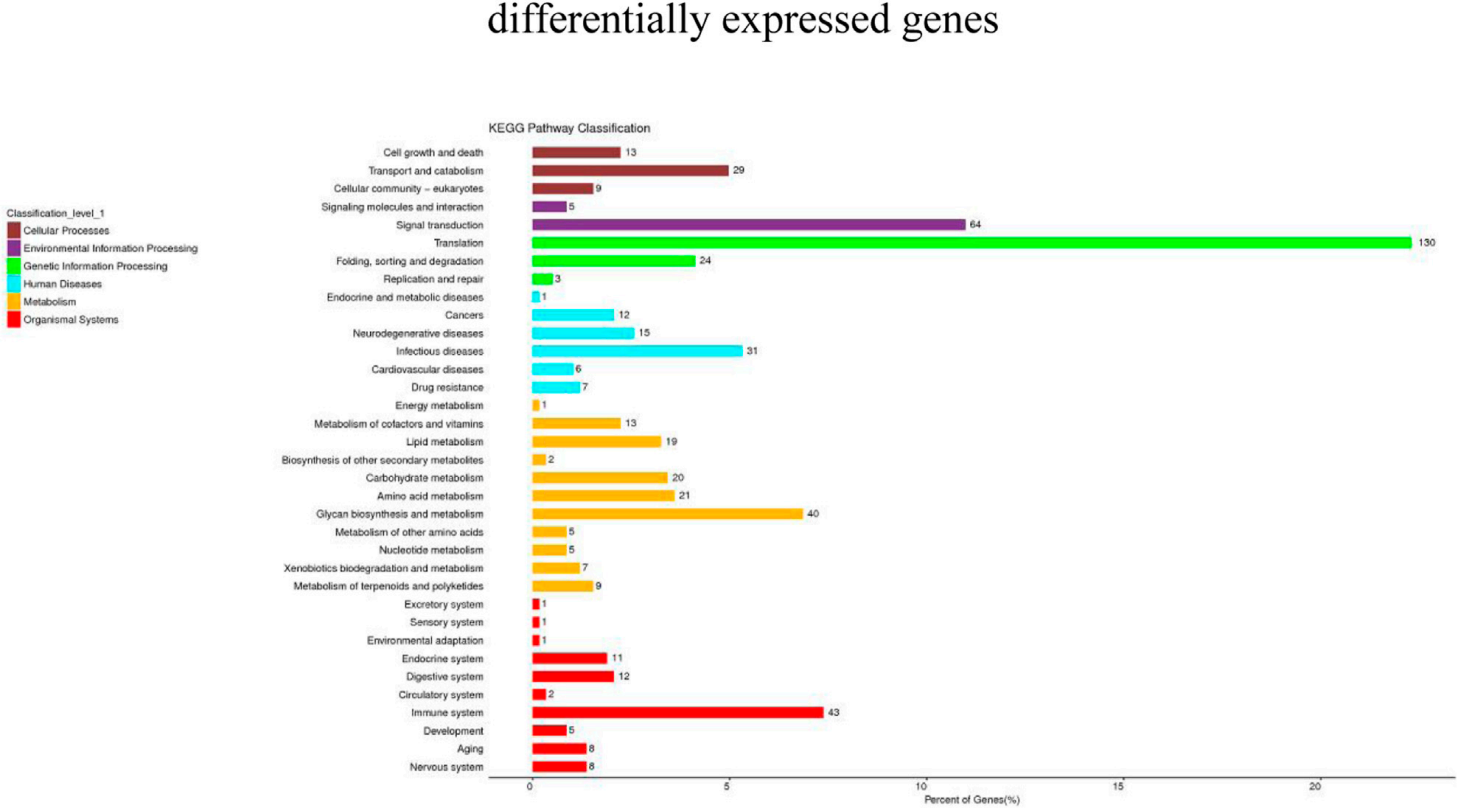
Statistical chart of KEGG Level 2 classification of DEGs.

**FIGURE 6:**
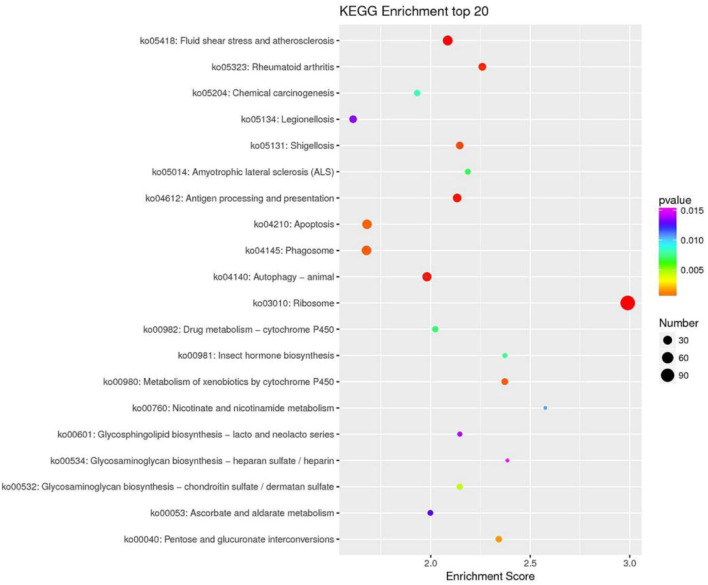
KEGG enrichment top 20 bubble plot (LC vs. HC).

KEGG pathway analysis can provide detailed information on the pathways in which DEGs are involved. In this study, 1,223 DEGs were annotated into 36 pathways. These pathways were divided into six main groups at Level 1: Metabolism, genetic information processing, environmental information processing, cellular processes, biological systems, and human diseases (Figure 5). There were significant differences in the enriched pathways between the experimental groups. In the HC vs. LC group, 583 DEGs significantly mapped to 37 signaling pathways. In the HC vs. MC group, 444 DEGs were significantly mapped to 28 signaling pathways. These pathways were mainly significantly associated with infectious diseases, signal transduction, immunity and metabolism. The top twenty pathways enriched in each group are shown in Figure 6.

### 3.6 Metabolic and immune-related differentially expressed genes

To investigate the effects of low salinity on the metabolic regulation of *P. monodon*, gill tissues were screened for metabolism-related DEGs after 45 days of stress with different salinities. Twelve metabolic pathways were identified, involving processes in amino acid metabolism, glucose metabolism and lipid metabolism. Five significantly enriched metabolic pathways were detected in the LC vs. HC group, including pentose and glucuronate interconversions, ascorbate and aldarate metabolism, cutin, suberin and wax biosynthesis, and sulfur metabolism. Three significantly enriched metabolic pathways were detected in the MC vs. HC group, including cutin, suberin and wax biosynthesis, and butanoate. Some representative differentially expressed genes are shown in Table 3.

**TABLE 3:** Partial metabolic differential genes.

Gene-id	Gene name	Gene function	Up/Down LC vs. HC
comp52003_c0_seq1_2	ATPeF1B	F-type H + -transporting ATPase	Down
CL1Contig15989	TPS	trehalose 6-phosphate synthase/phosphatase	Down
CL2932Contig1	NMT	phosphoethanolamine N-methyltransferase	Down
CL1Contig691	GPAT1_2	glycerol-3-phosphate O-acyltransferase 1/2	Up
CL1Contig18242	MLL1	histone-lysine N-methyltransferase MLL1	Up
CL7374Contig1	DHDH	dihydrodiol dehydrogenase/D-xylose 1-dehydrogenase	Down
CL1Contig14912	E3.1.3.56	inositol-1,4,5-trisphosphate 5-phosphatase	Up
CL1Contig3700	HMGCL, hmgL	hydroxymethylglutaryl-CoA lyase	Up
CL1Contig18298	ADPGK	ADP-dependent glucokinase	Up
CL11158Contig1	EHMT	euchromatic histone-lysine N-methyltransferase	Up
CL7586Contig1	ENPP1_3, CD203	ectonucleotide pyrophosphatase/phosphodiesterase family member 1/3	Up
CL1Contig18253	ASH1L	histone-lysine N-methyltransferase ASH1L	Up
CL1Contig14071	Gad	glutamate decarboxylase	Up
CL4166Contig1	ODC1	ornithine decarboxylase	Down

To investigate the effect of chronic low salinity on immune regulation in *P. monodon*, the study analyzed immune system pathways. A total of 17 immune system regulatory signaling pathways were detected, including the NOD-like receptor signaling pathway, Toll-like receptor signaling pathway, and Toll and IMD signaling pathways. The study detected more differentially expressed genes in the NOD-like receptor signaling pathway and Toll and IMD signaling pathways. In addition, antigen processing and presentation were significantly different only in the LC vs. HC group, and cell differentiation was only different in the MC vs. HC group. Some representative differentially expressed genes are shown in Table 4.

**TABLE 4:** Partial immune system differential genes.

Gene-id	Gene name	Gene function	Up/Down LC vs. HC
CL9263Contig1	HSP70s	heat shock 70 kDa protein 1/2/6/8	Down
CL582Contig1	htpG	molecular chaperone HtpG	Down
CL13706Contig1	CTSL	cathepsin L	Down
CL16147Contig1	UBE2D	ubiquitin-conjugating enzyme E2 D	Down
CL9717Contig1	trxA	thioredoxin 1	Down
CL16002Contig1	GRK4_5_6	G protein-coupled receptor kinase	Up
CL3197Contig1	FBXW1_11	F-box and WD-40 domain protein	Up
CL1953Contig2	GSN	gelsolin	Down

### 3.7 Signal transduction-related differentially expressed genes

Understanding signal transduction pathways is an important guide for resolving the adaptation mechanisms of organisms to environmental changes. Therefore, the signal transduction pathways of *P. monodon* under chronic low salinity stress were analyzed during this study. A total of 31 signal transduction pathways were identified, including the PI3K-Akt signaling pathway, MAPK signaling pathway, AMPK signaling pathway, Hippo signaling pathway, calcium signaling pathway, sphingolipid signaling pathway, cGMP-PKG signaling pathway, HIF-1 signaling pathway, cAMP signaling pathway and Ras signaling pathway. Among them, the MAPK signaling pathway was significantly different in both the LC vs. HC and MC vs. HC groups, while the Hippo signaling pathway was only significantly different in the MC vs. HC groups. Some representative differentially expressed genes are shown in Table 5.

**TABLE 5:** Partial signal transduction differential genes.

Gene-id	Gene name	Gene function	Up/Down LC vs. HC
CL1Contig15487	BMP4	bone morphogenetic protein 4	Down
CL1Contig18178	SIAH1	E3 ubiquitin-protein ligase SIAH1	Up
CL6915Contig1	MAPK1_3	mitogen-activated protein kinase 1/3	Up
comp10606_c0_seq1_2	XPO1	exportin-1	Up
CL13099Contig1	ndk, NME	nucleoside-diphosphate kinase	Down
CL16049Contig1	ADIPOR	adiponectin receptor	Up
CL1Contig20063	RAPTOR	regulatory associated protein of mTOR	Up
CL1Contig20760	SCD	stearoyl-CoA desaturase (Delta-9 desaturase)	Up
CL1Contig9488	MDM2	E3 ubiquitin-protein ligase Mdm2	Up
comp77726_c0_seq4_3	BRCA1	breast cancer type 1 protein	Up
CL1Contig13091	EX	protein expanded	Up
CL1Contig791	MOB1	MOB kinase activator 1	Up
CL11393Contig1	PIK3C2	phosphatidylinositol-4-phosphate 3-kinase	Up
CL1Contig16972	ABL1	abelson tyrosine-protein kinase 1	Up
comp26341_c0_seq1_2	MCU	calcium uniporter protein, mitochondrial	Up
CL1Contig12468	PPP1R12A	protein phosphatase 1 regulatory subunit 12A	Up
comp3209_c1_seq1_2	KCNMA1	calcium-activated channel subfamily M alpha member 1	Down
CL15441Contig1	ARF1	ADP-ribosylation factor 1	Down
CL1Contig2700	CIC	capicua transcriptional repressor	Up
CL13825Contig1	YWHAE	14-3-3 protein epsilon	Down
comp41280_c0_seq1_2	RPS6	small subunit ribosomal protein S6e	Down
CL2913Contig1	ACTB_G1	actin beta/gamma 1	Up
CL15002Contig1	CAMK2	calcium/calmodulin-dependent protein kinase (CaM kinase) II	Down
CL14773Contig1	SLC25	solute carrier family 25	Down

## 4 Discussion

Although previous studies have shown that *P. monodon* can tolerate a wide range of salinities, studies on its osmotic adaptation mechanisms under chronic low salinity stress are still lacking. The mechanisms of adaptation of *P. monodon* to long-term varying salinity were assessed by full-length transcriptome amplification in our study. This study identified 2,062 DEGs from the HC vs. MC group, including 1,000 upregulated and 1,062 downregulated genes, and identified 2,671 DEGs from the HC vs. LC group, including 1,140 upregulated and 1,531 downregulated genes. This difference in gene regulation suggests that although *P. monodon* can cope with this stress, 18 psu and 3 psu may cause subtle stress. The GO analysis results are consistent with previous studies on aquatic animals that classified DEGs into three categories of biological processes, molecular functions and cellular components, such as *P. vannamei* (Yu et al., 2022), *Pelteobagrus fulvidraco* (Wang et al., 2022) and Oratosquilla oratoria (Lou et al., 2019). Furthermore, KEGG analysis showed that changes in salinity significantly affected metabolic, immune and signaling pathways in *P. monodon*, consistent with the results of transcriptome analyses of *P. vannamei* (Chen et al., 2015), *Acanthogobius ommaturus* (Sun et al., 2020) and *Eriocheir sinensis* (Yang et al., 2019) under low salinity stress. Therefore, the present study focused on the adaptation of metabolic, immune and signal transduction mechanisms in relation to salinity.

Salt stress is largely responsible for altering the metabolic state of organisms (Sun et al., 2020; Liu Z. et al., 2021). Our study found that the expression of metabolism-related genes was significantly affected and showed metabolic imbalance in *P. monodon* during prolonged low salinity stress. Many studies have reported similar results, but most of the studies on metabolism in osmoregulation have focused on osmotic physiological responses (Guo et al., 2020). In the present study, three significantly enriched metabolic pathways were detected in the MC vs. HC group, including Cutin, suberin and wax biosynthesis, Butanoate metabolism and Valine, leucine and isoleucine biosynthesis; five significantly enriched metabolic pathways were detected in the LC vs. HC group, including Pentose and glucuronate interconversions, Ascorbate and aldarate metabolism, Cutin, suberin and wax biosynthesis, Sulfur metabolism, Arginine biosynthesis and Phenylalanine metabolism. These pathways are mainly related to the regulation of glucose, lipid and amino acid metabolism. It has been found that crustaceans exposed to salinity fluctuations require additional energy to ensure osmotic pressure regulation and ion exchange (Chen et al., 2014). Lipids may provide sufficient energy to maintain ion homeostasis and regulate the permeability of membrane structures and therefore may play an important role in osmotic pressure regulation (Palacios et al., 2004; Tseng and Hwang, 2008). Free amino acids are osmotic effectors in crustaceans, and the metabolism of free amino acids may provide energy and eventually lead to cell growth in crustaceans (Lemos et al., 2001). Thus, metabolic pathways play an important role in the regulation of osmotic pressure in crustaceans.

Chronic low salinity stress can damage the immune system of aquatic animals. Antigen processing and presentation and the NOD-like receptor signaling pathway are the immune pathways that are more affected by chronic low salinity stress in *P. monodon*. Antigen processing and presentation are the cornerstones of adaptive immunity, through which organisms immunologically recognize some bacteria, viruses and parasites (Pishesha et al., 2022). Therefore, changes in genes associated with this pathway suggest that chronic low salinity stress may disrupt the innate immunity of shrimp and increase their susceptibility to bacteria, viruses, and parasites, among others (Kelly and Trowsdale, 2019). Complement and coagulation cascades are evolutionarily relevant blood enzyme cascade reactions in the blood. The complement system has immunoprotective and regulatory functions, while the coagulation cascades are responsible for ensuring hemostasis, and together, they limit pathogen infection through innate immune mechanisms. Thus, both protein C (PROC) and plasminogen (PLG), important regulators in the complement and coagulation cascades in this study, were significantly upregulated, suggesting that prolonged hyposalinity stress may have some effect on coagulation function in *P. monodon* (Ayón-Núñez et al., 2018). In addition, the NOD-like receptor signaling pathway (NLR) immune recognition factor was significantly altered in both groups. This regulation has important implications for NLR and complement components, which are pattern recognition receptors that recognize molecular patterns associated with viral, bacterial and eukaryotic pathogens (Ward and Rosenthal, 2014). For crustaceans, the innate immune system plays a crucial role in defense against microbial infections and environmental stress (Tran et al., 2022). These results suggest that *P. monodon* may have an increased susceptibility to pathogen attack in low salinity environments.

The adaptive and stimulatory responses of aquatic animals to salt stress depend on effective mechanisms of osmotic sensing and osmotic stress signaling (Nie et al., 2017). Furthermore, osmotic sensing signals are not only rapidly transduced intracellularly but may also be amplified and distributed to multiple downstream osmotic effects (Sun et al., 2020). cAMP, as a signaling factor, is involved in the regulation of osmotic pressure by mediating the increased uptake of Na^+^ by cells in the internal environment (Liang et al., 2022). At the same time, cAMP stimulates the production of calmodulin (CaM) and ATP, thereby regulating stress, apoptosis and calcium homeostasis (Huang et al., 2020). Thus, as important ion transport regulatory proteins, CaM and NKA are significantly upregulated at low salinity. The PI3K-AKT pathway is one of the most actively studied kinase pathways, mediating cell growth, survival, cell cycle progression, differentiation, transcription, translation, and glucose metabolism in animals (Liu et al., 2018). Therefore, downregulation of this pathway may be responsible for the slow growth of *P. monodon* under prolonged hyposaline conditions, as shrimp require more energy for osmoregulation and ion homeostasis during normal growth (Cui et al., 2020). The MAPK pathway is important for osmotic pressure regulation, and chilling and hypoxia are important. In the present study, DEGs in the gill tissue of *P. monodon* under salt stress were enriched in the MAPK signaling pathway, indicating that the pathway was induced. Similar results were found in *P. vannamei* hemocytes under salt stress (Zhao et al., 2015). Furthermore, AMPK is suitable as an intracellular energy and pressure sensor to reveal the molecular mechanisms underlying temperature, hypoxia and salinity fluctuations in animals (Hardie, 2011). Salinity stress can temporarily activate AMPK, while glucose depletion leads to sustained AMPK activation. Moreover, in both cases, AMPK activation levels depend on the degree of stress (Schutt and Moseley, 2017). The activation of the AMPK signaling pathway and the expression of related genes in *P. monodon* under low salinity stress may be due to its continuous activation by glucose depletion. The differences in the expression of genes related to the AMPK signaling pathway in gill tissues of *P. monodon* at different salinities may be due to different salt stress stresses, resulting in different energy regulation mechanisms.

## 5 Conclusion

In this study, RNA-seq analysis of *P. monodon* exposed to three salinity gradients (namely, HC, MC and LC) was carried out, and the high-quality data obtained are the best guarantee of experimental reliability. These changes were verified by this experiment to be related to metabolic, immune system and signal transduction properties. Thus, *P. monodon* may adapt to changes in environmental salinity by regulating mechanisms such as metabolism, immunity and signal transduction. These data will provide basic data for future selection and breeding for resistance in the *P. monodon* industry, which will contribute to the sustainable and healthy development of shrimp farming.

## Data Availability

The datasets presented in this study can be found in online repositories. The names of the repository/repositories and accession number(s) can be found below: https://www.ncbi.nlm.nih.gov/, SRR11805651, SRR11805650, SRR11805649, SRR11805654, SRR11805653, SRR11805652, SRR11805665, SRR11805664, and SRR11805655.
